# Development of a Janus nanofibrous patch with antibacterial and anti-oxidative properties for urethral regeneration

**DOI:** 10.7150/thno.112435

**Published:** 2025-05-25

**Authors:** Wang Wang, Zesheng Chen, Ruoyu Li, Zhijun Zhou, Guanyi Wang, Xinjun Su, Weikang Hu, Zijian Wang, Xingyuan Xiao, Bing Li

**Affiliations:** 1 Department of Urology, Zhongnan Hospital of Wuhan University, Wuhan 430071, China; 2 Department of Biomedical Engineering, Hubei Province Key Laboratory of Allergy and Immune Related Disease, TaiKang Medical School (School of Basic Medical Sciences), Wuhan University, Wuhan 430071, China; 3 School of Materials Science and Engineering, Stem Cells and Tissue Engineering Manufacture Center, Hubei University, Wuhan 430062, China

**Keywords:** MXenes, nanofiber, antibacterial, anti-oxidative, urethral regeneration

## Abstract

**Background:** Urethral injury is the primary cause of urinary tract stenosis and hydronephrosis. Limited by the common drawbacks of autografts, the clinical treatment of urethral injury remains challenging. In recent years, biocompatible and biodegradable biomaterials (BBBs) are emerging as a potential substitute for autografts to upgrade the research paradigms of regenerative medicine. However, ideal BBBs for urethral regeneration have rarely been reported.

**Methods:** A Janus nanofibrous PC/SMC patch composed of the outer layer of PLLA/CRRI-3 nanofibers and the inner layer of SF/MCe heterojunction nanofibers were first fabricated. Its antibacterial and antioxidant properties were assessed. After passing biosafety evaluation, the patch's efficacy in repairing urethral defects was evaluated using a rabbit model, with repair outcomes analyzed via histological staining.

**Results:** PC/SMC patch not only inhibits bacterial proliferation and survival via the release of the antibacterial peptide CRRI-3, but it also relieves oxidation stress and promotes tissue regeneration by the nanozyme-like activities of the MCe heterojunction. The biocompatibility of PC/SMC patch has met the general requirements for Class-III medical devices. The application *in vivo* was evaluated using a urethral injury model of rabbits. The results showed that PC/SMC patch could improve urethral regeneration and prevent urethral stricture via multiple mechanisms, including promoting re-epithelialization, cell proliferation and M2 macrophage polarization, and inhibiting of fibrosis and scar formation.

**Conclusion:** The PC/SMC nanofibrous patch has good biocompatibility and antibacterial properties, and can effectively promote the regeneration and repair of urethral tissue.

## Introduction

Urethral injury is a common emergency caused by mechanical shock, sexual activity, inflammation and iatrogenic factors 1, 2. The anatomy of urethral is complicated including urethral epithelium, blood vessels, nerves and submucosal tissue 3. Urethral injury can lead to numerous complications, including urinary tract infections, urine extravasation, and urethral fistulas 4. Urethral stricture is the most severe complication of urethral injury, with an incidence rate ranging from 200 - 1,200 cases per 100,000 individuals 5. Urethral stricture is pathologically characterized by the disorder of extracellular matrix (ECM) and fibrosis 6. A series of risk factors, including bacterial infections, oxidative stress and corrosion of urine, can exacerbate this process. In the past decades, plastic and reconstructive surgery (PRS) of urinary tract has made significant progresses to solve these challenges. For example, a wide variety of autografts, such as lingual mucosal, appendix, flap, mucosa grafts and ileum, have been utilized as patch materials to repair the urethra 7-10. In our group, it was proved that the recovery rate of lingual mucosal has reached to 97.6%.

Harvesting autologous tissue patches would causes secondary trauma 11. Thus, artificial tissue-engineered materials for defect repair have drawn much attention in the field of reconstructive surgery. For instance, small intestinal submucosa (SIS) has been reported in a few case reports for urethral reconstruction. But in patients with complex urethral stricture, its long-term restenosis and urinary tract reconstruction fistula rates are relatively high 12. In addition, bacterial cellulose (BC), a naturally derived polymeric patch from *bacillus lignocellulosus*, is highly explorable in urethral reconstruction thanks to its good biocompatibility, high mechanical strength, and unique structure 13. However, its poor biodegradability greatly restricts its practical application in urethral repair 14. Thus, developing novel, multifunctional tissue-engineered materials suited for the urethral environment and applying them to urethral repair is highly significant.

Electrospun nanofibers have been widely explored in the field of regeneration medicine including wound healing, bone and nerve repair, but are rarely reported in urethral injury 15-17. Due to the ECM-like structure, electrospun nanofibers can promote the adhesion, proliferation and migration of host cells, thus to improve the regeneration effect 18-20. In a previous review, our group has summarized a series of electrospun nanofibers with desirable biocompatibility and biodegradability 21. Among them, poly-L-lactic acid (PLLA) nanofibers and silk fibroin (SF) nanofibers are more superior. PLLA nanofibers are mechanically tough, and hydrophobic to resist urine and external risk factors 22. SF nanofibers are flexible and biocompatible, making them more suitable to function as a tissue engineering scaffold 23, 24. Here, it is assumed that the PLLA nanofibers and SF nanofibers can be integrated into a Janus nanofibrous patch. Specifically, PLLA nanofibers serve as the outer layer of urethral, and SF nanofibers serve as the inner layer of urethral. Such a Janus nanofibrous patch is designed to adapt to the wound microenvironment (WME) of urethral 25, 26. To better meet the clinical needs, the broad-spectrum antibacterial and anti-oxidative activities of Janus nanofibrous patch remains to be improved.

CeO_2_ nanoparticles are anti-oxidative nanozymes with CAT-like and SOD-like activities 27, 28. CeO_2_ nanoparticles have recently emerged as a potent tool for modulating the oxidative stress state of WME and improving wound healing 29, 30. A series of modification strategies have been proposed to improve the anti-oxidative activity of CeO_2_ nanoparticles. For example, Hu et al. reported a Ti_3_C_2_/CeO_2_ (MCe) heterojunction 31, 32. Ti_3_C_2_ nanosheets are typical MXenes, also known as 2D transition metal carbides, nitrides, and carbonitrides 33, 34. The formation of Ti_3_C_2_/CeO_2_ heterojunction could significantly reduce the energy band, and improve the catalytic rate compared to neat CeO_2_ nanoparticles. Herein, it is assumed that Ti_3_C_2_/CeO_2_ heterojunction can be incorporated into SF nanofibers to endow the Janus nanofibrous patch with pro-longed anti-oxidative activity. Antibacterial peptides CRRI-3, with a peptide sequence of REERWF-NH_2_, is first reported by Huang et al. in 2023 35. With a help of artificial intelligence (AI), CRRI-3 was screened out from a virtual library with a data set of 64 million. Compared to naturally derived peptides, CRRI-3 is chemically synthesized, offering low cost, good solubility, better antibacterial activity and non-toxicity 36-38. Thus, it is assumed that CRRI-3 can be incorporated into PLLA nanofibers to endow the Janus nanofibrous patch with enhanced broad-spectrum anti-oxidative activity.

**Figure 1:**
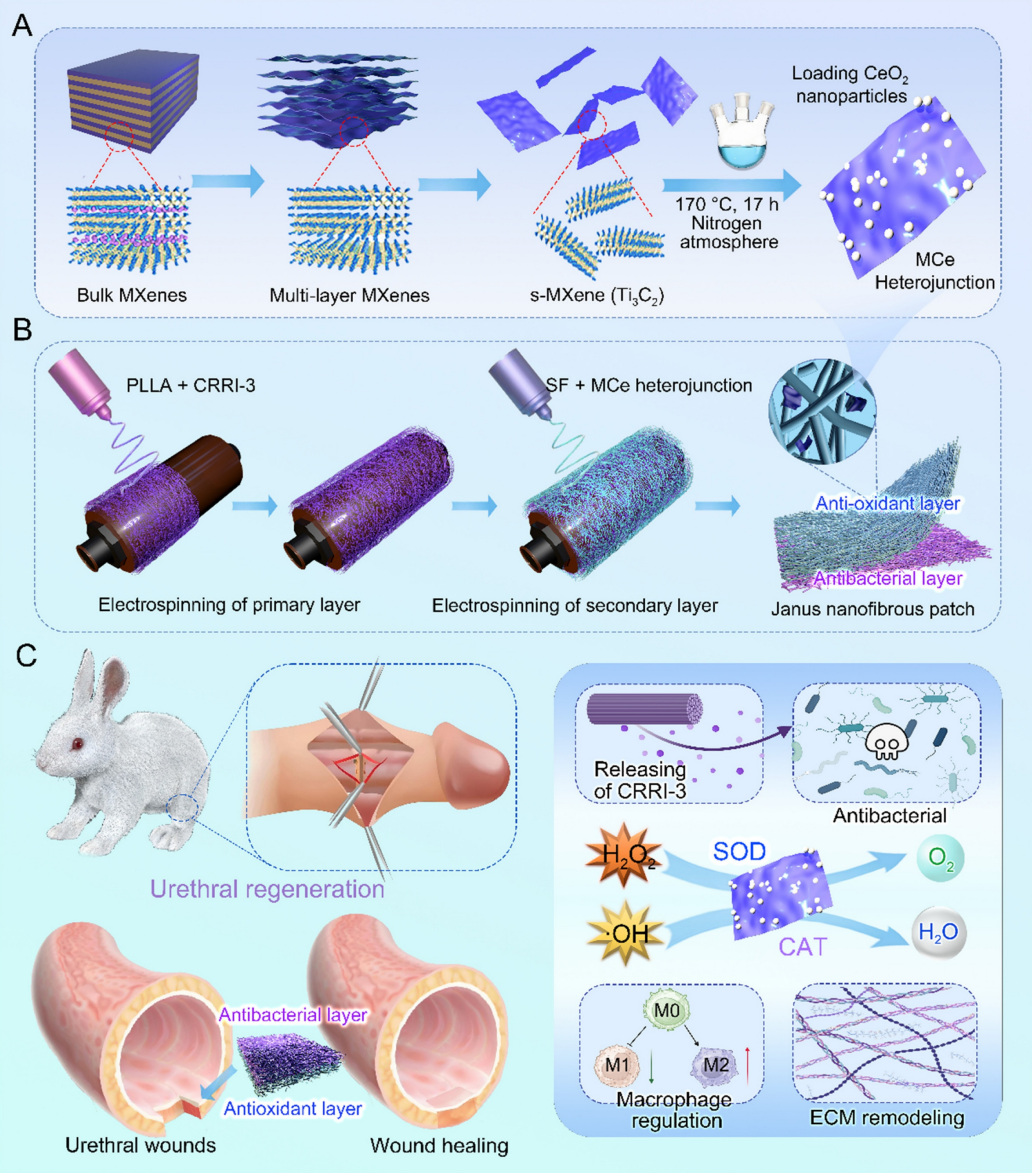
** The preparation and biomedical applications of Janus nanofibrous PC/SMC patch.** (**A**) The synthesis process of MXenes/CeO_2_ (MCe) heterojunction, including chemical etching and hydrothermal reaction; (**B**) Janus nanofibrous patch was prepared using a two-step electrospinning method; (**C**) Janus nanofibrous patch was successfully applied for urethral regeneration. The potential bioactivities and mechanisms were highlighted.

This study designed a Janus nanofibrous patch composed of an outer layer of PLLA/CRRI-3 nanofibers and an inner layer of SF/MCe heterojunction nanofibers to improve urethral regeneration. A diagram of this study is shown in **Fig. 1**. MCe heterojunction was synthesized using a hydrothermal reaction. After that, MCe heterojunction was mixed with SF solution, and CRRI-3 was mixed with PLLA solution, respectively. A two-step electrospinning was employed to fabricate the Janus nanofibrous patch which was coded as PC/SMC patch. The physiochemical properties, antibacterial and anti-oxidative activities, and biocompatibility of PC/SMC patch will be comprehensively investigated. Finally, the pro-regenerative effect and mechanisms of PC/SMC patch will be explored using a urethral injury model of rabbits. This study will provide a promising alternative of autografts for urethral scar-free regeneration.

## Experimental Methods

### Preparation of MCe heterojunction

#### Etching of monolayer Ti_3_C_2_ nanosheets

Monolayer Ti_3_C_2_ nanosheets were obtained using an improved chemical etching method. Briefly, 1 g of bulk MAX was dispersed in a mixture of 20 mL of hydrofluoric acid, 20 mL of hydrochloric acid and 10 mL of distilled.

The mixture was magnetically stirred at 37 °C for 12 h, and then washed with distilled water until the pH value reached to 7.0. After centrifugation at 3000 rpm for 5 min, the precipitate was dispersed into 2% LiCl solution and stirred at 37 °C for another 12 h. The mixture was sonicated in an ice bath for 30 min to prepare monolayer Ti_3_C_2_ nanosheets, which were subsequently freeze-dried and stored at room temperature.

#### Preparation of MCe composite heterojunction

Ti_3_C_2_/CeO_2_ (MCe) heterojunction were synthesized using a hydrothermal method. Briefly, 30 mg of Ti_3_C_2_ nanosheets were added into 30 mL of distilled water and dispersed by ultrasonic for 30 min. After that, 10 mg of Ce(NO_3_)_3_·6H_2_O and 2 mL of 25% NH_3_·H_2_O were dissolved in the mixture and reacted at 170 °C for 17 h. After centrifugation at 3000 rpm for 5 min, the precipitate was washed with anhydrous ethanol for three times, and then freeze-dried for further study.

### Purification of silk fibroin

Silkworm cocoons were cut into pieces, and the raw silk was soaked in a 0.5% sodium carbonate solution at 100 °C for 30 min to degum. This degumming process was repeated twice, followed by washing with distilled water for three times. The silk was dissolved in a ternary solvent, stirred at 70 °C for 1 h, and dialyzed using distilled water for 3 days. The filtered solution was freeze-dried to obtain silk fibroin (SF).

### Preparation of Janus nanofibrous patch

Two kinds of electrospinning solution were prepared 39. Briefly, 1.0 g of PLLA was dissolved in 10 mL of 1,1,1,3,3,3-hexafluoro-2-propanol (HFIP) and stirred overnight. Antimicrobial peptide CRRI-3 was added to obtain a PLLA/CRRI-3 solution (coded as PC solution). The concentration of CRRI-3 was set to 0.1%. Meanwhile, 1 g of SF was dissolved in 10 mL of HFIP, and an appropriate amount of MCe heterojunction was added to obtain a SF/MCe solution (coded as SMC solution).

As previously reported, the Janus nanofibrous patch was prepared using an electrospinning method. The flow rate was set to 1 mL/h, the voltage was set to 9 kV, the electrospinning time was at least 4 h. The collector plate was maintained at a distance of 12 cm from the positive electrode. In this study, six kinds of nanofibrous materials, including PLLA nanofibers, PC nanofibers, SF nanofibers, SMC nanofibers, Janus PC/SMC patch and P/S patch were prepared using the same parameters.

### Anti-oxidative evaluations

#### Free radical scavenging

The efficiency of PC/SMC patch in scavenging free radicals was evaluated using a DPPH assay. DPPH working solution was prepared by dissolving 1 mg of DPPH in 20 mL of anhydrous ethanol. DPPH solution (1 mL), anhydrous ethanol solution (0.1 mL), and SF, SMC, and PC/SMC samples (100 mg) were incubated at 37 °C in the dark place for 8 h. After centrifugation, the absorbance of supernatant was detected using a UV-Vis spectrophotometer. The DPPH**·** scavenging rate was calculated using the following formula:



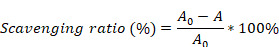



where A_0_ represents the absorbance of blank control (DPPH**·** + ethanol), and A irepresents the absorbance of experimental group (DPPH**·** + SF, SMC, PC/SMC).

The efficiency of PC/SMC patch was also evaluated using an oxTMB assay. oxTMB solution was prepared by dissolving TMB (0.25 mM), H_2_O_2_ (50 mM) and FeCl_2_·4H_2_O (1 mM) in citric acid/sodium citrate buffer (pH = 4.5). oxTMB solution (1 mL) was incubated with deionized H_2_O (0.1 mL), and SF, SMC, and PC/SMC samples (100 mg) at 37 °C in the dark place for 8 h. After centrifugation, the absorbance of supernatant was detected. The oxTMB scavenging rate was calculated using the following formula:



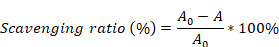



where A_0_ represents the absorbance of blank control (oxTMB + ddH_2_O), and A represents the absorbance of experimental group (oxTMB + SF, SMC, PC/SMC).

#### Flow Cytometry for detecting ROS

L929 cells were seeded onto a 6-well plate and cultured until they reached 80% confluence. After that, 100 µM of H_2_O_2_ and SMC nanofibers were added. After 24 h of incubation, the cells were detected using a ROS detection kit according to the manufacturer's instructions. The cell proliferation was also detected by CCK-8 assay according to standard protocols.

#### Cell viability and migration assay

HUVEC and L929 cells were treated with 100 µM of H_2_O_2_ and a piece of nanofibers for 24 h. After that, a live/dead cell staining assay was performed according to the manufacturer's instructions. As previously reported, the migration ability of treated cells was evaluated using a scratching assay 40. Optical images were captured using a laser confocal microscope.

### Broad-spectrum antibacterial evaluations

*E. coli* and *S. aureus* were selected as the representatives of gram-negative and gram-positive bacteria, respectively. Single colony from bacterial agar plate was picked and activated in Luria-Bertani (LB) liquid medium overnight. The activated bacterial suspension was co-cultured with PC/SMC patch. According to our previous report 41, the broad-spectrum antibacterial activity of PC/SMC patch was evaluated by bacterial proliferation assay and clone formation assay at the same time. A live/dead bacterial staining assay was performed according to the manufacturer's instructions. The nanofibrous materials were collected, washed with PBS for three times and fixed with paraformaldehyde for 2 h. The bacteria adhered onto the surface of materials were dehydrated and observed using a SEM.

### Urethral Defect Model Establishment and Surgical Repair

The experimental protocol was approved by the Institutional Animal Care and Use Committee (IACUC) of Wuhan University's Animal Experiment Center (approval no. WP20230604). Twenty-four male New Zealand rabbits, weighing about 2.5 kg, were purchased from Slike Jingda Lab Animal Co., Ltd. (Hunan, China). These animals were randomly divided into four groups (n = 6). The animals were anesthetized with pentobarbital, and the hair around the urethra was shaved. A full-thickness urethral defect was created approximately 2 cm from the external urethral orifice, with an average length × width of 1 cm × 0.5 cm. The urethral lumen was exposed, and a 6-0 absorbable suture was used to suture. Control group was directly sutured; SIS group was sutured with commercial SIS; P/S group and PC/SMC group was sutured with P/S patch and PC/SMC patch, respectively.

Urethral contrast examinations of living animals were performed at postoperative 5 and 10 weeks. After the retrograde urethral contrast, the rabbits were euthanized. Urethral tissues were collected for histological analysis, including Hematoxylin and Eosin (H&E) staining and Masson's staining. These tissues were fixed with 4% paraformaldehyde at room temperature for 3 days, followed by dehydration and paraffin embedding. To further demonstrate the repair effect, the samples were also subjected to a series of immunofluorescence (IF) staining, including AE1/AE3, cell Ki67, CD86, CD206, and TGF-β. According to the manufacturer's protocols, the relative expression of IL-6 and TNF-a at Week 5 was also detected by ELISA. Primary antibody and secondary antibody used in this study are listed in **Table S1** and **Table S2**. Fluorescence intensity was quantified using Image J software.

### Statistical Analysis

The statistical significance of all experiments was determined using one-way analysis of variance (ANOVA) and paired sample t-tests. Error bars represent the mean ± standard deviation (SD). A difference was considered statistically significant at *P* < 0.05 (* *P* < 0.05, ** *P* < 0.01, *** *P* < 0.001). *n.s.* indicates no significant difference.

## Results and Discussion

### MCe heterojunction with desirable anti-oxidative property was synthesized

**Figure 2:**
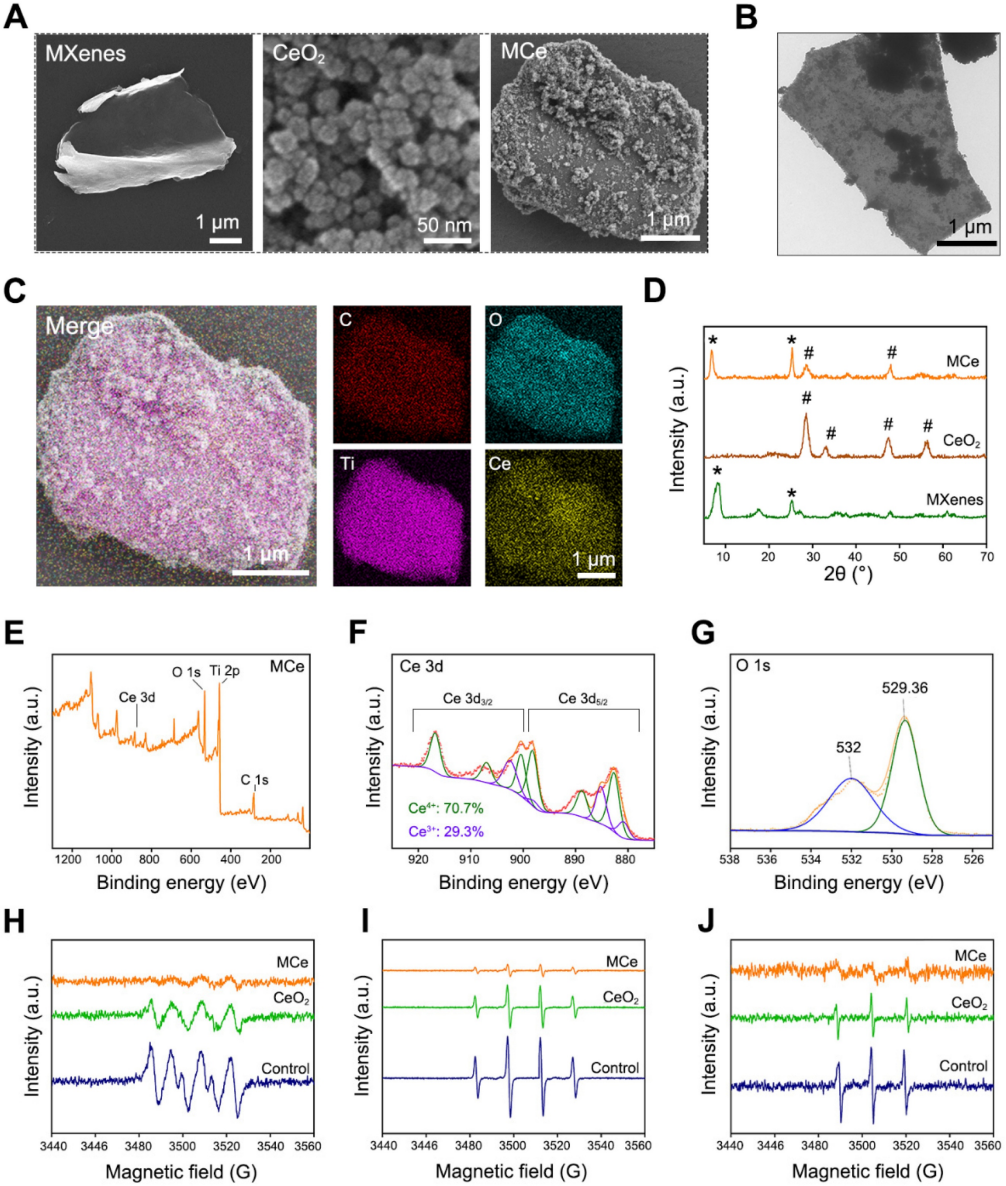
** Preparation and characterizations of MXenes/CeO_2_ (MCe) heterojunction.** (**A**) SEM images of monolayer MXenes, CeO_2_ nanoparticles and MCe heterojunction. Scale bar: 1 µm or 50 nm; (**B**) TEM images of MCe heterojunction. Scale bar: 1 µm; (**C**) SEM mapping of MCe heterojunction, showing the element components. Scale bar: 1 µm; (**D**) XRD spectrum of monolayer MXenes, CeO_2_ nanoparticles and MCe heterojunction; (**E-G**) XPS spectrum of MCe heterojunction; (H) EPR analysis for detecting superoxide anion; (**I**) EPR analysis for detecting hydroxyl radicals; (**J**) EPR analysis for detecting singlet oxygen.

In this study, a novel heterojunction material based on monolayer MXenes (namely Ti_2_C_3_) and CeO_2_ nanoparticles was designed. The structure of heterojunction was supposed to endow the products with enhanced catalytic activity. As shown in **Fig. 2A**, monolayer MXenes at micrometer scale were successfully exfoliated from bulk MAX using an improved etching method 42. Additionally, CeO_2_ nanoparticles and composite heterojunction (namely MCe) were synthesized using a hydrothermal method 43. CeO_2_ nanoparticles evenly distributed onto the surface of MCe heterojunction, which is consistent with the results of TEM observation in **Fig. 2B**. SEM mapping was performed to verify the element compositions of MCe heterojunction. As shown in **Fig. 2C**, the O element and Ce element on the outer surface of MCe heterojunction were marked with blue and yellow, respectively. The relative contents were 23.58% for C element, 35.58% for O element, 31.03% for Ti element, and 9.81% for Ce element. These results indicated that MCe heterojunction was successfully synthesized. XRD was performed to characterize the crystalline structure of MCe heterojunction. As shown in **Fig. 2D**, MXenes exhibited two peaks at 6.71° and 25.2°. These characteristic peaks could be found in MCe group. MCe heterojunction also exhibited two characteristic peaks of CeO_2_ nanoparticles located at 28.5° and 47.4°. However, these characteristic peaks at 33.1° and 56.7° disappeared. This phenomenon suggested that the crystalline structure of CeO_2_ nanoparticles was partially destroyed during the synthesis processes of MCe heterojunction. Notably, the defect engineering of MCe heterojunction will make a positive impact on the biomedical applications. For example, accelerated catalytic rate and fast releasing of ceria ions.

XPS was performed to analyze the valence state of MCe heterojunction. As shown in **Fig. 2E-G** and **Fig. S1A-B**, MCe heterojunction exhibited four characteristic peaks including Ce 3d, O 1s, Ti 2p and C 1s. In particular, Ce element had both Ce^4+^ and Ce^3+^ phenotypes with a ratio of 70.7: 29.3. Ce^3+^ and Ce^4+^ are the most important bioactive factors of CeO_2_-based nanozymes. Researchers have proved that the POD-like activity of CeO_2_-based nanozymes is achieved through reversible conversion between Ce^3+^ and Ce^4+^ 8. The potential mechanisms include but are not limited to: 1) catalyzing H_2_O_2_ and lipid peroxide (LPO) to generate hydroxyl radical (·OH); 2) Electron transfer occurs between the substrate and H_2_O_2_ 44, 45. Thus, the MCe heterojunction obtained in this study had anti-oxidative potential. Herein, a series of EPR analysis were performed the verify this assumption. As shown in **Fig. 2H-J**, MCe heterojunction could eliminate superoxide anion, hydroxyl radical and singlet oxygen, confirming desirable anti-oxidative activity. Notably, the anti-oxidative activity of MCe heterojunction was significantly better than that of CeO_2_ nanoparticles. This phenomenon was attributed to the formation of heterojunction structure.

### A nanofibrous patch with Janus structure and properties was prepared

**Figure 3:**
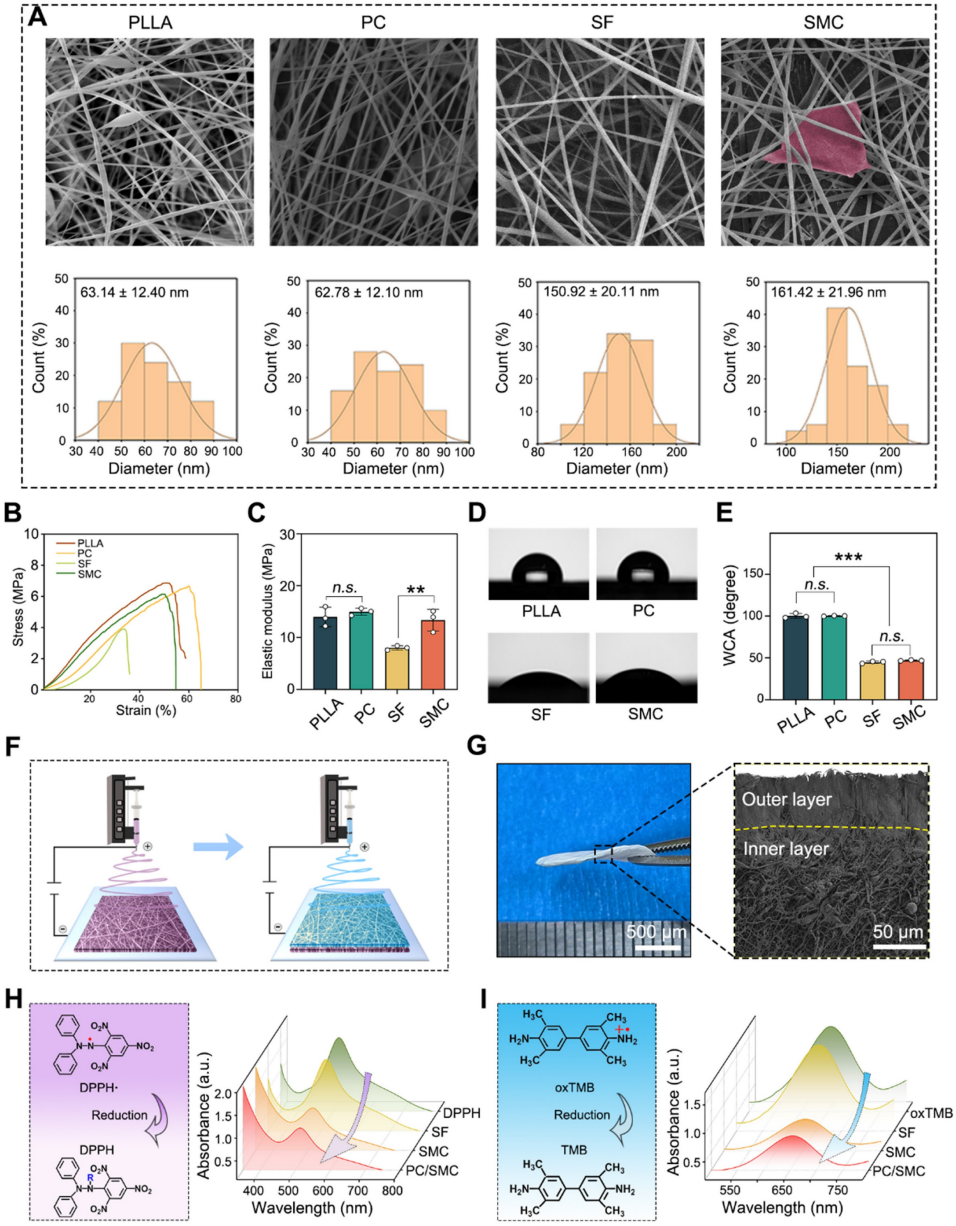
** Preparation and characterizations of Janus nanofiber patch (PC/SMC).** (**A**) SEM images of four kinds of electrospun nanofibers, including neat PLLA nanofiber, PLLA/CRRI-3 (P/C) composite nanofiber, neat SF nanofiber and SF/MCe (SMC) composite nanofiber. The diameter distribution of each nanofiber was counted. Scale bar: 2 µm; (**B-C**) Tensile test of the nanofibers (n = 3); (**D-E**) Water contact angle (WCA) test (n = 3); (**F**) A diagram showing the preparation process of Janus nanofibrous patch; (**G**) Morphological observation of Janus nanofibrous patch (PC/SMC); (**H-I**) Anti-oxidative evaluations, including DPPH assay and TMB assay. Values are shown as the Mean ± SD. For the significant comparison, *n.s.* indicates no significance, ***P < 0.001.

Electrospun nanofibers are suitable for urethral regeneration because of their mechanical strength, flexibility, biocompatibility and biodegradation 46, 47. However, conventional nanofibers could not fulfill the clinical needs of urethral regeneration, such as broad-spectrum antibacterial and anti-oxidation. Thus, the structure and properties of nanofibrous materials needs to be modified. Herein, four kinds of nanofibers were prepared using an electrospinning method. As shown in **Fig. 3A**, all materials exhibited typical 3D network structure, suggesting that the electrospinning parameters used in this study were appropriate. The average diameter was 63.14 ± 12.4 nm for PLLA group, 62.78 ± 12.1 nm for PLLA/CRRI-3 (PC) group, 150.92 ± 20.11 nm for SF group, 161.42 ± 21.96 nm for SF/MCe (SMC) group, respectively. Antibacterial peptide CRRI-3 was added into PC nanofibers to endow them with antibacterial activity, and MCe heterojunction was added into SMC nanofibers to improve their anti-oxidative activity. The tensile strength and hydrophilicity of these nanofibers were detected. As shown in **Fig. 3B-C**, the elastic modulus was 13.98 ± 1.89 MPa for PLLA group, 14.98 ± 0.68 MPa for PC group, 8.01 ± 0.42 MPa for SF group and 13.36 ± 2.10 MPa for SMC group, respectively. The incorporation of MCe heterojunction has significantly improved the mechanical strength of SMC nanofibers (*P* < 0.001). As shown in **Fig. 3D-E**, the WCA was 100.25 ± 2.89° for PLLA group, 100.17 ± 0.38° for PC group, 44.85 ± 1.34° for SF group and 46.91 ± 0.52° for SMC group. PC nanofibers are more hydrophobic than SMC nanofibers (*P* < 0.001). Thus, PC nanofibers are more advantages in terms of resisting urine and hydrophilic toxins.

Based on the properties of PC and SMC nanofibers, a nanofibrous patch with Janus structure was designed. **Fig. 3F** shows the preparation process of Janus nanofibrous patch. The outer layer was composed of PC nanofibers, which are mechanical tough and hydrophobic to resist urine and hydrophilic toxins, and play a vital role in combating bacterial infection via sustained releasing of antibacterial peptide 48. The inner layer was composed of SMC nanofibers, which relieve oxidative stress by the POD-like activity of MCe heterojunction, and function as a tissue engineering scaffold to improve the adhesion, proliferation and migration of host cells 49. As shown in **Fig. 3G**, the Junus nanofibrous patch was successfully prepared and coded as PC/SMC patch. As shown in **Fig. 3H-J** and **Fig. S2A-B**, PC/SMC patch had good POD-like activities. It was concluded that the preparation processes of Janus structure did not disturb the biochemical properties of each layer.

### PC/SMC patch has good anti-oxidative activity

**Figure 4:**
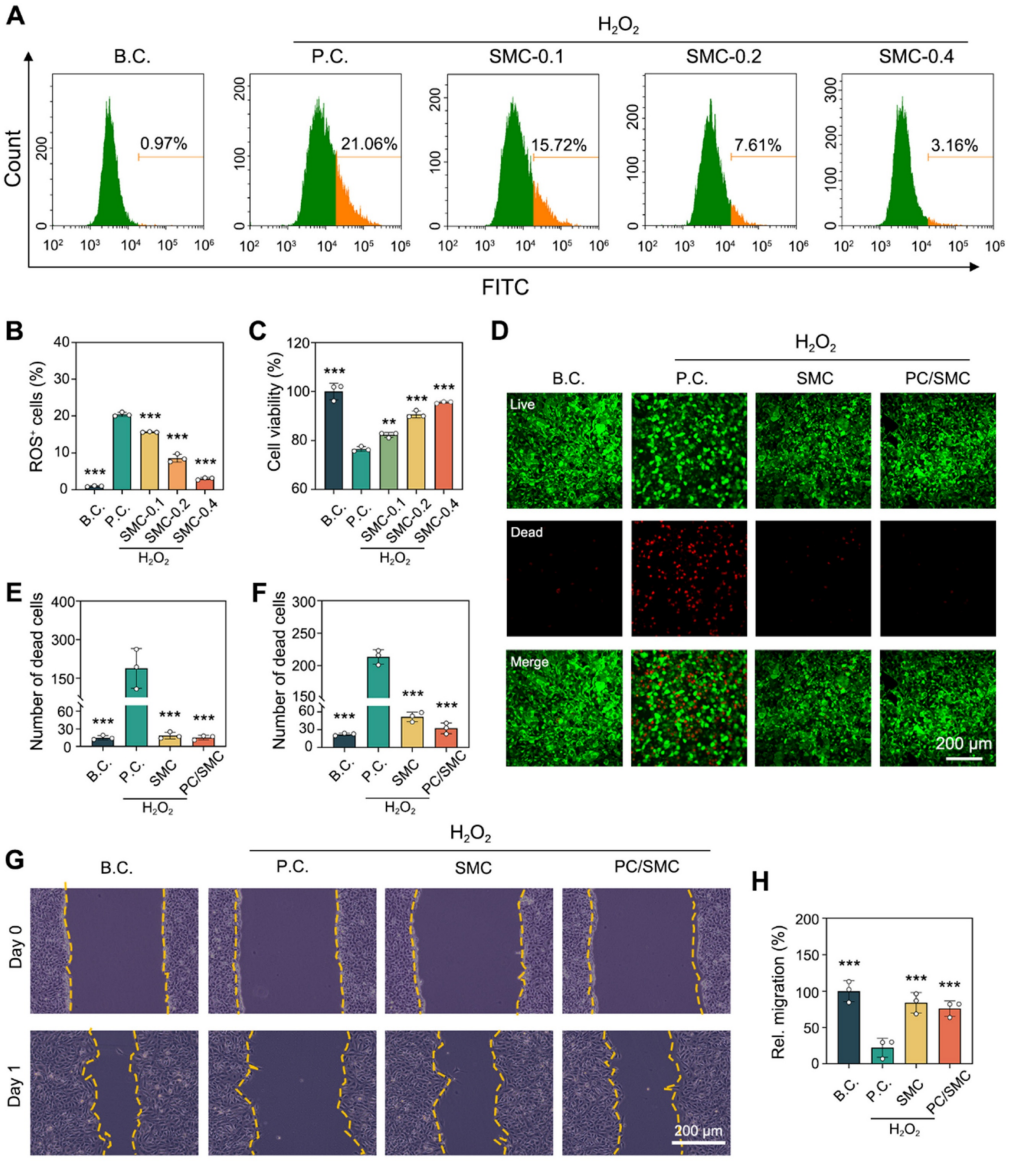
** Anti-oxidative evaluations using a H_2_O_2_-induced model of oxidative stress injury.** (**A-B**) Flow cytometry for detecting intercellular ROS level (n = 3); (**C**) CCK-8 assay for detecting cell viability (n = 3); (**D**) Live/dead cell staining images of L929 cell. Scale bar: 200 µm; (**E**) Number of dead L929 cells per field (n = 3); (**F**) Number of dead HUVEC cells per field (n = 3); (**G-H**) The migration ability of HUVEC cells were evaluated by scratching assay (n = 3). Values are shown as the Mean ± SD. Compared to P.C. group, **P < 0.01, ***P < 0.001.

As previously reported, the anti-oxidative activity of SMC nanofibers and PC/SMC patch was evaluated using a cell-based model 48. As shown in **Fig. 4A-B**, L929 cells were co-cultured with 100 µM of H_2_O_2_ solution to induce oxidative stress injury. After that, the cells were treated with the materials for 48 h. The percentage of ROS positive cells was 0.97 ± 0.15% for B.C. group, and that was 20.43 ± 0.56% for P.C. group. This phenomenon suggested that the cell-based model of oxidative stress was successfully established. The percentage of ROS positive cells was 15.61 ± 0.09% for SMC-0.1 group, 8.58 ± 1.10% for SMC-0.2 group, and 3.02 ± 0.31% for SMC-0.3 group. Compared to P.C. group, significant differences were observed (*P* < 0.001). It could be concluded that the anti-oxidative activity of SMC nanofibers had a typical dose-dependent effect. As shown in **Fig. 4C**, the anti-oxidative activity of SMC nanofibers could significantly rescue the viability of H_2_O_2_-treated cells (*P* < 0.01).

The incorporation of anti-oxidative SMC nanofibers was supposed to endow the PC/SMC patch with enhanced bioactivity. Herein, a live/dead cell staining assay was performed to visualize this assumption. The results of L929 cells are shown in **Fig. 4D-E**. Live cells were dye green and dead cells were dye red. The number of dead cells per field was 15 ± 3.6 for B.C. group, 225.3 ± 51.6 for P.C. group, 18.7 ± 5.7 for SMC group, and 15.3 ± 3.8 for PC/SMC group. P.C. group exhibited most dead cells among four groups. The results of HUVEC cells are shown in **Fig. 4F** and **Fig. S3**, and exhibited similar trend. A scratching assay was performed to evaluate the cell migration ability. The results of HUVEC cells are shown in** Fig. 4G-H**. After 24 h of incubation, the relative migration rate was 100 ± 14.4% for B.C. group, 22.1 ± 13.4% for P.C. group, 84 ± 14% for SMC group and 76 ± 10.6% for PC/SMC group, respectively. Compared to P.C. group, significant differences were observed (*P* < 0.001). The results of L929 cells are shown in **Fig. S4A-B**, and exhibited similar trend. In conclusion, SMC nanofibers and PC/SMC patch could both rescue the survival and migration of host cells in oxidative stress state, thus making an active pro-regeneration impact on wound healing 50, 51.

### PC/SMC patch has good broad-spectrum antibacterial activity

The incorporation of PC nanofibers was supposed to endow the PC/SMC patch with broad-spectrum antibacterial activity.

**Figure 5:**
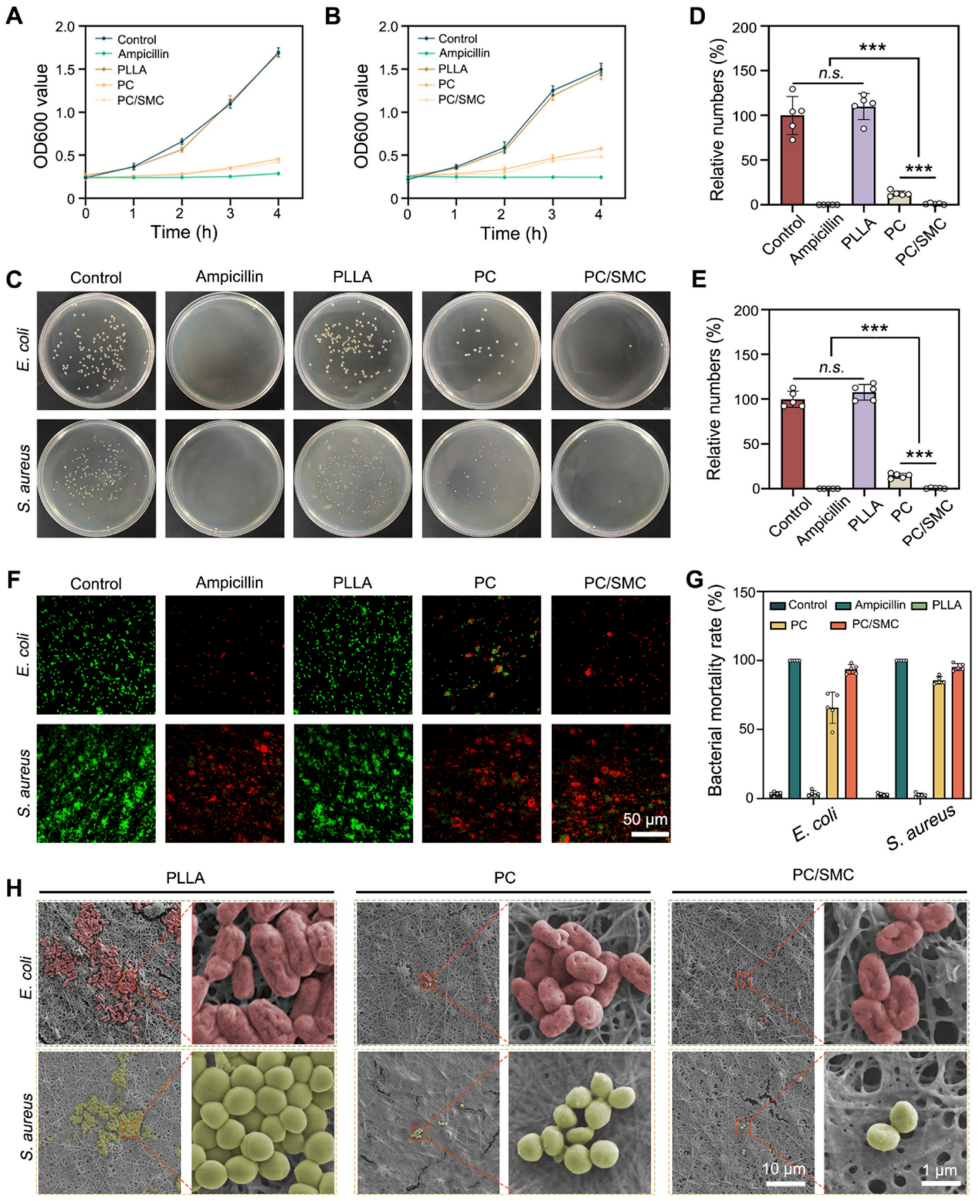
** Broad-spectrum antibacterial evaluations *in vitro*.** (**A**) Bacterial proliferation curves of *E. coli* bacteria; (**B**) Bacterial proliferation curves of *S. aureus* bacteria; (**C**) Images of bacterial clones; (**D-E**) Relative numbers of bacterial clones in both *E. coli* and *S. aureus* bacteria (n = 5); (**F**) Live/dead bacterial staining images of both *E. coli* and *S. aureus* bacteria. Scale bar: 50 µm. (**G**) Quantitative results of bacterial mortality rate (n = 5); (**H**) SEM images of *E. coli* and *S. aureus* bacteria in varied groups. Scale bar: 10 µm or 1 µm. Values are shown as the Mean ± SD. For the significant comparison, *n.s.* indicates no significance, ***P < 0.001.

Herein, the broad-spectrum antibacterial activity was comprehensively evaluated using two kinds of model organisms. The results of bacterial proliferation assay are shown in **Fig. 5A-B**. PC nanofibers and PC/SMC patch could effectively inhibit the proliferation rate of both *E. coli* and *S. aureus*. As shown in **Fig. 5C-E**, the survival ability of these treated bacteria was evaluated. For *E. coli* bacteria, the relative numbers of bacteria clone were 100 ± 21.2% for control group, 0 ± 0% for ampicillin group, 110 ± 14.5% for PLLA group, 12.5 ± 3.0% for PC group and 1.1 ± 0.9% for PC/SMC group; for *S. aureus* bacteria, those were 107.8 ± 8.8% for PLLA group, 14.5 ± 2.7% for PC group and 0.59 ± 0.61% for PC/SMC group, respectively. PLLA nanofibers are not antibacterial, and the antibacterial activity of PC nanofibers and PC/SMC patch are mainly attributed to the releasing of antibacterial peptide CRRI-3.

A live/dead bacterial staining assay was performed to visualize the bacterial survival state. As shown in **Fig. 5F**, live bacteria were dye green, and dead bacteria were dye red. The quantitative results of live/dead bacterial staining assay are shown in **Fig. 5G**. More than 70% of bacteria in ampicillin group, PC group and PC/SMC group was dye red, indicating good antibacterial effect. As shown in **Fig. 5H**, the bacterial cells adhered onto the outer surface of nanofibrous materials exhibited varied morphology. In particular, the bacterial cells in PC and PC/SMC groups obviously shrunk compared to those in PLLA group. These results furtherly confirmed the good broad-spectrum antibacterial activity of PC nanofibers and PC/SMC patch.

### PC/SMC patch has compliant compatibility

**Figure 6:**
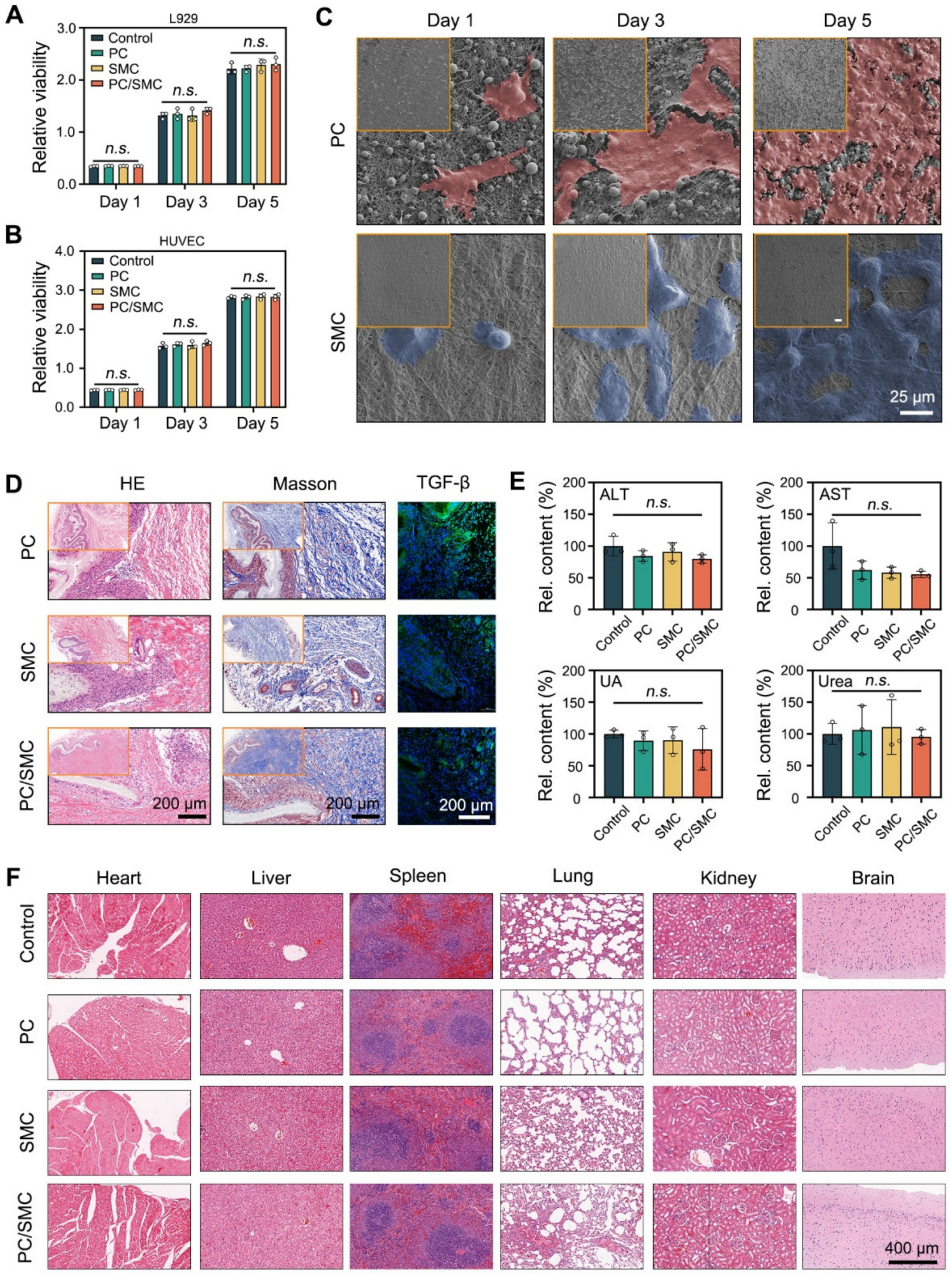
** Biocompatibility evaluations *in vitro* and* in vivo*.** (**A-B**) Relative viability of L929 cells and HUVEC cells co-cultured with different nanofibers (n = 3); (**C**) Morphological observations of L929 cells adhered onto the surface of PC and SMC nanofibers. Scale bar: 35 µm; (**D**) Histological staining images after transplanted *in vivo* for 15 days. Scale bar: 200 µm; (**E**) Blood biochemical tests, including Alanine Aminotransferase (ALT), Aspartate aminotransferase (AST), uric acid (UA) and urea (n = 3); (**F**) H&E staining images of six kinds of organs resected from the treated animals. Scale bar: 400 µm. Values are shown as the Mean ± SD. For the significant comparison, *n.s.* indicates no significance.

In this chapter, the biocompatibility of PC/SMC patch and its' raw materials was comprehensively evaluated. For evaluations* in vitro*, L929 cells and HUVEC cells were co-cultured with their extracts for 5 days. After that, the viability of these cells was detected by CCK-8 assay (**Fig. 6A-B**). At each time point, the relative viability among four groups exhibited no significant difference (*P* > 0.05). These results indicated that PC/SMC patch won't disturb cell proliferation. As shown in **Fig. 6C**, HUVEC cells were seeded onto PC nanofibers (the outer layer of PC/SMC patch) and SMC nanofibers (the inner layer of PC/SMC patch) for 5 days. These cells adhered evenly onto the surface of materials, and the number of cells gradually increased along with an increase of culturing time. In conclusion, PC/SMC patch could support cell adhesion, and served as a tissue engineering matrix to induce regeneration. Compared to PC group, the cells in SMC group exhibited more typical cell morphology. Thus, SMC nanofibers are more suitable to be the inner layer of PC/SMC patch which requires better cytocompatibility.

As previously reported, a subcutaneous transplantation model of SD rats was established to evaluate the biocompatibility of PC/SMC patch *in vivo* 52. The materials were transplanted for 15 days. The materials along with the capsule tissue were then resected for a series of histological analysis. As shown in** Fig. 6D**, capsule tissue formed around the materials, owning to the immune rejection reaction. These materials did not degrade within 15 days, which was complaint with the clinical needs of urethral regeneration. Furthermore, the capsule tissue was mainly composed of collagen with less inflammatory cells infiltrated, suggesting that the histocompatibility of these materials was relatively good. Researchers have proved that transforming growth factor beta (TGF-b) plays a vital role in modulating collagen remodeling and scar formation 53. Meanwhile, scar formation ranks one of the most important causes of postoperative urethral and ureter stricture 54. In this study, the expression of TGF-b in PC/SMC group was at a moderate level. Thus, it was inferred that PC/SMC patch is less likely to generate scar formation and urethral restenosis.

Fresh whole blood of these treated animals was collected for biochemical tests. The results are shown in **Fig. 6E** and **Fig. S5A-F**. Compared to control group (Sham-operated), each index in other groups exhibited no significant difference (*P* > 0.05). The organs of the treated animals were also resected for H&E staining analysis. As shown in **Fig. 6F**, the heart, liver, spleen, lung, kidney and brain tissues in four groups did not exhibit typical pathological changes. In conclusion, the biocompatibility of PC/SMC patch and the raw materials could meet the needs of medical devices for applications* in vivo*.

### PC/SMC patch significantly promote urethral regeneration

**Figure 7:**
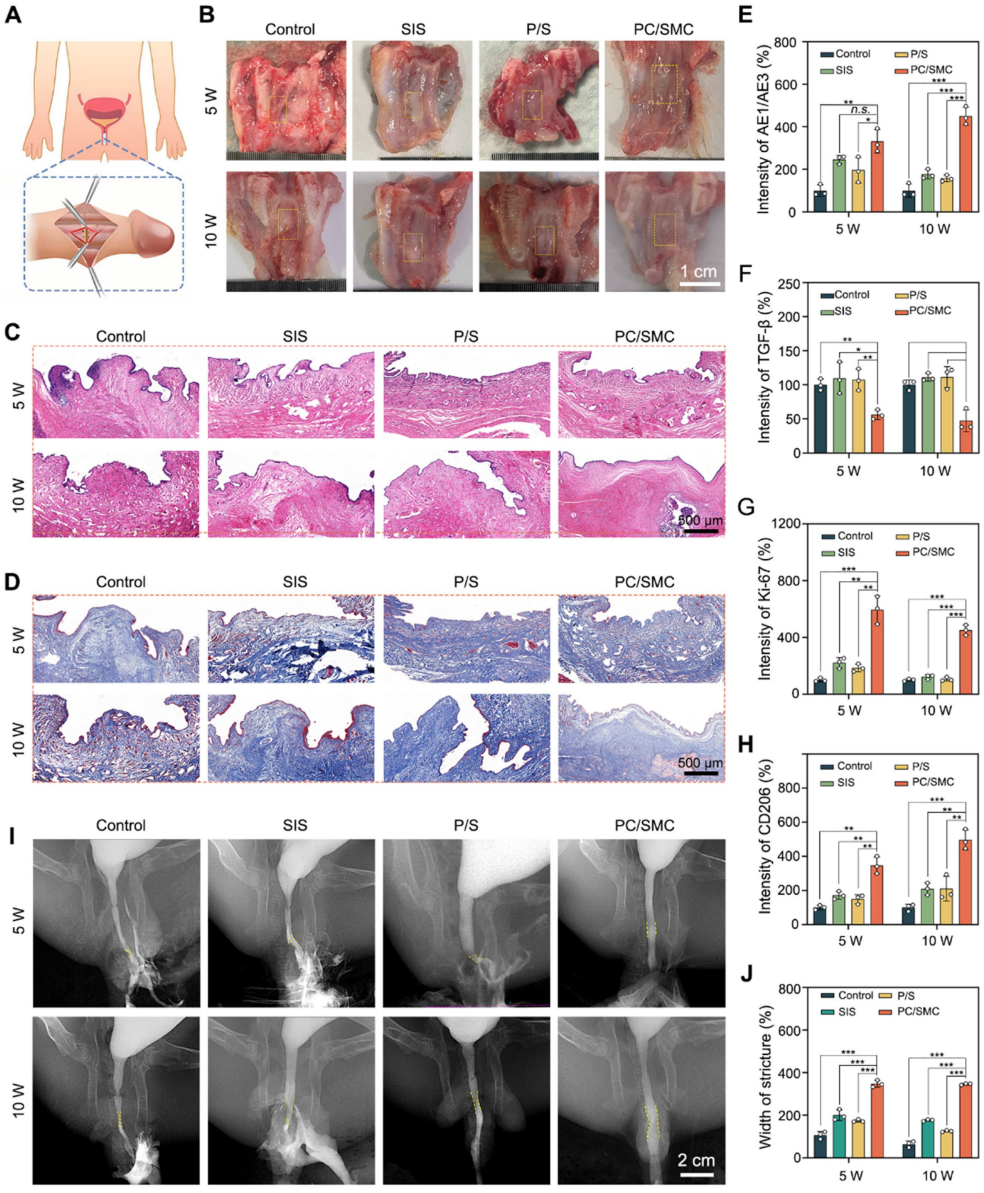
** Urethral regeneration evaluations.** (A) A diagram showing the surgical operations; (B) Optical images of neo-urethral tissue. Scale bar: 1 cm; (C) H&E staining images. Scale bar: 500 µm; (D) Masson's staining images. Scale bar: 500 µm; (E-H) Quantitative results of a series of histological markers, including AE1/AE3, TGF-b, Ki-67 and CD206 (n = 3); (I) X-ray urethrography. Scale bar: 2 cm; (J) Relative width of structure (n = 3). Values are shown as the Mean ± SD. Compared to PC/SMC group, *n.s.* indicates no significance, *P < 0.05, **P < 0.01, ***P < 0.001.

The biomedical applications of PC/SMC patch were preliminarily explored using a urethral injury model of New Zealand rabbits. As shown in **Fig. 7A**, a surgical incision was created in the mid-section of penis of each animal, and immediately sutured with a piece of PC/SMC patch. The control group were end to end sutured, the SIS group was sutured with a commercial product based on decellularized extracellular matrix, and P/S group was sutured with PLLA/SF patch without bioactive MCe heterojunction and CRRI-3. After conventionally fed for 10 weeks, neo-urethral tissue formed among four groups (**Fig. 7B**). It was concluded that injured urethral could repair and regenerate to restore normal anatomical structures and physiological functions to a certain extent. However, some patients in clinical practice may experience urethral stricture, which is the main challenge. Thus, it is important to evaluate the regeneration quality from richer dimensions.

Neo-urethral tissue was used for a series of histological analysis. The results of H&E staining assay are shown in **Fig. 7C**. At 5 weeks after surgery, loose granulation tissue formed in four groups. A large number of neo-blood vessels were also found, which is beneficial for oxygen and nutrition supply. At 10 weeks after surgery, PC/SMC group exhibited complete mucosal layer, including keratinized epithelium and subcutaneous tissue, while other groups did not. This phenomenon suggested that the regeneration rate of PC/SMC group was faster than that of other groups. The results of Masson's staining assay are shown in **Fig. 7D**.

PC/SMC group exhibited the lowest expression of collagen, showing less possibility of urethral stricture. The potential mechanisms could be partially attributed to its' biocompatibility, broad-spectrum antibacterial and anti-oxidative properties.

As shown in **Fig. 7E-H** and **Fig. S6-10**, PC/SMC patch could significantly up-regulate the expression of AE1/AE3, Ki-67 and CD206, and down-regulated the expression of CD86, TGF-b, IL-6 and TNF-a. These results showed that PC/SMC could promote the re-epithelialization, cell proliferation and macrophage polarization of neo-urethral tissue, and inhibit fibrosis and scar formation. Thus, the pre-regenerative mechanisms of PC/SMC patch were very diverse. Herein, an X-ray urethrography was performed to visualize the urethral stricture in living animals. As showing in **Fig. 7I-J**, PC/SMC groups exhibited biggest width of stricture among four groups. Compared to PC/SMC group, significant differences were observed (*P* < 0.001). Based on the above results, we found that the overall application effect of PC/SMC patch was significantly better than that of SIS. Thus, the PC/SMC patch has exhibited great potential for clinical translation.

This study developed a Janus nanofibrous PC/SMC patch composed of PLLA/CRRI-3 nanofibers and SF/MCe heterojunction. The PC/SMC patch has good broad-spectrum antibacterial and anti-oxidative activities and biocompatibility, and could improve urethral regeneration and avoid urethral stricture via multiple mechanisms. The PC/SMC patch is expected to replace traditional autografts, such as lingual mucosal and appendix, with bright prospects for clinical translation 55, 56. Despite the promising results of the PC/SMC patch in the rabbit urethral defect model, our study has certain limitations. The rabbit urethral model, while offering a valuable experimental environment with anatomical and physiological similarities to the human urethra, exhibits differences in defect size and severity compared to human urethral diseases. For future research, we plan to validate our findings in other animal models, such as minipigs, to enhance the reliability of the results.

## Conclusions

The work developed a Janus nanofibrous PC/SMC patch with desirable broad-spectrum antibacterial and anti-oxidative activities for effective urethral regeneration. The outer layer of PC/SMC patch was composed of PLLA/CRRI-3 nanofibers, which are mechanical tough and hydrophobic to resist urine and hydrophilic toxins, and play a vital role in combating bacterial infection via sustained releasing of antibacterial peptide. The inner layer was composed of SF/MCe heterojunction nanofibers, which relieve oxidative stress and function as a tissue engineering scaffold to improve tissue regeneration. The findings demonstrated that PC/SMC patch met the needs of Class-III medical devices for applications *in vivo*. PC/SMC patch could also improve urethral regeneration and avoid urethral stricture via diverse mechanisms, including promotion of re-epithelialization, cell proliferation and macrophage polarization, and inhibition of fibrosis and scar formation. The efficacy of PC/SMC patch has surpassed that of commercial SIS patch. The Janus nanofibrous PC/SMC patch as a promising alternative to autografts can be used not only for urethral regeneration, but also for bladder and ureter regeneration.

## Supplementary Material

Supplementary figures and tables.
