# Supplementary material for: Development of a Janus nanofibrous patch with antibacterial and anti-oxidative properties for urethral regeneration
Source: Theranostics. 2025 May 25;15(13):6428–43. doi: 10.7150/thno.112435 (PMC12160026; doi:10.7150/thno.112435)
Supplement: Supplementary file 1 — Supplementary figures and tables. [file thnov15p6428s1.pdf]

Supplementary information

Development of a Janus nanofiber patch with antibacterial and anti-oxidative properties for urethral regeneration

Wang Wang[1,2*], Zesheng Chen[1,3*], Ruoyu Li[1], Zhijun Zhou[1], Guanyi Wang[1], Xinjun Su[1], Weikang Hu[3], Zijian Wang[1,2✉], Xingyuan Xiao[1✉] and Bing Li[1✉]

1. Department of Urology, Zhongnan Hospital of Wuhan University, Wuhan 430071, China
2. Department of Biomedical Engineering, Hubei Province Key Laboratory of Allergy and Immune Related Disease, TaiKang Medical School (School of Basic Medical Sciences), Wuhan University, Wuhan 430071, China
3. School of Materials Science and Engineering, Stem Cells and Tissue Engineering Manufacture Center, Hubei University, Wuhan 430062, China

* These authors contributed equally to this work.

✉ Corresponding authors: Department of Urology, Zhongnan Hospital of Wuhan University, Wuhan 430071, China. E-mail address: Zijianwang@whu.edu.cn (Z. Wang); xiaoxingyuan@whu.edu.cn (X. Xiao); bingli2023@whu.edu.cn (B. Li).

Materials

Poly-L-lactic acid (PLLA) was purchased from Haishan Technology Co., Ltd. (Wuhan, China). Bulk MAX (Ti_3AlC_2) was obtained from XFNANO Material Tech. Co., Ltd. (Nanjing, China). $Ce(NO_3)_3\cdot 6H_2O$, LiCl, HCl, hydrofluoric acid, 25% $NH_3\cdot H_2O$, tryptone, agar, and yeast extract were acquired from Sinopharm Chemical Reagent CO., Ltd. (Shanghai, China). 2,2-Diphenyl-1-picrylhydrazyl (DPPH), 3,3',5,5'-Tetramethylbenzidine (TMB), 5,5-Dimethyl-1-pyrroline N-oxide (DMPO), and 2,2,6,6-Tetramethyl-4-piperidone monohydrate (TEMP) were purchased from Aladdin Bioengineering Co. Ltd. (Shanghai, China). Dulbecco's Modified Eagle Medium (DMEM) with high glucose, fetal bovine serum (FBS), 0.25% trypsin, and phosphate-buffered saline (PBS) were acquired from Pricella Biotechnology Co., Ltd. (Wuhan, China). The Live/dead bacterial staining kit and the calcein-AM/PI double stain kit were sourced from Yeasen Biotechnology Co., Ltd. (Shanghai, China). Reactive oxygen species (ROS) detection kit was purchased from Beyotime Biotechnology Co., Ltd. (Shanghai, China). Silkworm cocoons were purchased from Jiaxing Silk Co., Ltd. (Jiangsu, China). Antimicrobial peptide CRRI-3, with the amino acid sequence REERWF-NH2, was synthesized and identified by Sangon Biotech Co., Ltd. (Shanghai, China). Other chemicals and reagents were used as received.

Materials characterizations

The morphology of Ti_3C_2 nanosheets, CeO_2 nanoparticles, MCe heterojunction, and composite nanofibers were observed using scanning electron microscopy (SEM). The diameter of nanofibers was statistically calculated using Image J sofeware. MCe heterojunction was dispersed into ethanol, dropped onto copper grids for morphological observation of transmission electron microscopy (TEM) at a voltage of 80 kV. The element composition of MCe heterojunction was analyzed using energy dispersive spectrometer (EDS). The surface chemical state and crystal structure of MCe heterojunction were detected using X-ray photoelectron spectroscopy (XPS) and X-ray diffraction (XRD), respectively.

Mechanical property testing

The nanofibers were cut into strips measuring 5 cm in length and 1 cm in width.

Tensile tests were conducted using a universal testing machine (UTM). The thickness of the nanofibrous membrane was measured using electronic calipers. The nanofiber strips were fixed in a clamp with an initial distance of 2 cm between the clamps. The clamp stretching speed was set at 2 mm/min. The stress-strain curves were recorded. At least three samples were used for statistical analysis.

Hydrophilicity test

The hydrophilicity of the nanofibers was evaluated using a contact angle measurement device (CAMD). Samples were fixed on a sample plate, and 10 μL of normal saline was dropped onto the upper surface of the nanofibers using a micro syringe. Photos were taken immediately, and the water contact angle (WCA) was measured using the goniometer method.

Electron paramagnetic resonance (EPR) analysis

For O^{2-} detection, methanol (100 μL), xanthine oxidase and xanthine were mixed and reacted for 5 min, followed with the addition of MCe and CeO_2 dispersions. After another 5 min, DMPO (250 mM) was added as a trapping agent. The solution was immediately transferred to a sample tube to record the EPR spectrum of O^{2-}.

For ·OH detection, double distilled water (ddH_2O, 50 μL), H_2O_2 (10 mM, 50 μL), and $FeCl_2$ (1 mM, 20 μL) were mixed and reacted for 5 min, followed by the addition of MCe and CeO_2 dispersions. After another 5 min, DMPO (250 mM) was added as a trapping agent. The solution was immediately transferred to a sample tube to record the EPR spectrum of ·OH.

For $^1O_2$ detection, ddH_2O (50 μL), H_2O_2 (10 mM, 50 μL), and sodium hypochlorite (NaClO) (10 mM, 20 μL) were mixed and reacted for 5 min, followed by the addition of MCe and CeO_2 dispersions. After another 5 min, TEMP (250 mM) was added as a trapping agent. The solution was immediately transferred to a sample tube to record the EPR spectrum of $^1O_2$.

Biocompatibility evaluations

Biocompatibility *in vitro*

L929 cells and HUVEC cells are cultured using DMEM high-glucose medium, and seeded onto a 96-well plate at a density of 1500 cells per well. The nanofibers were

added onto the medium and co-incubated at 37 °C for 5 days. At regular time intervals, the viability of cells was detected by CCK-8 assay. The cells are seeded onto the upper surface of PC nanofibers and SMC nanofibers for 5 days. At regular time intervals, the samples were collected and fixed with 4% paraformaldehyde for 30 min. The morphology of cells adhered onto the nanofibers was observed by SEM.

Biocompatibility *in vivo*

This study was conducted with the approval of the Animal Care and Welfare Committee of Zhongnan Hospital, Wuhan University (WP20230604). Twelve healthy SD rats, weighing 180-200 g, were purchased from Slike Jingda Lab Animal Co., Ltd. (Hunan, China). All SD rats were fed in a specific pathogen-free environment for 7 days to reduce stress. A subcutaneous implantation model was established. The samples were cut into pieces with a diameter of 1 cm. SD rats were anesthetized with 3% pentobarbital and disinfected with 0.5% iodine tincture. The skin on their back was incised. The subcutaneous connective tissue was freed to place the samples underneath. The skin was sutured, and the rats were conventionally fed. After 15 days of fed, fresh blood was collected from the rats using coagulation tubes, and plasma was obtained by centrifugation for immediate detection of a series of biochemical indicators. Additionally, the materials, heart, liver, spleen, lung, kidney and brain of the rats were collected for H&E staining assay.

Table S1. List of primary antibodies

**Antigens**	Species antibodies raised in	Dilution (IF)	Supplier
AE1/AE3,	Mouse,monoclonal	1:100	Santa Cruz Biotechnology, USA, cat. # SC-81714
TGF-β	Mouse, monoclonal	1:100	Novus Biologicals, USA, cat. # NBP2-46108
Ki67	Mouse, monoclonal	1:3000	Novus Biologicals, USA, cat. # NBP3-05537
CD206	Mouse, monoclonal	1:400	Proteintech Group, China, cat. # 60143-1-I
TGF-β	Rabbit, monoclonal	1:200	Bioss Biotechnology, China, cat. # bs-0086R
CD86	Rabbit, monoclonal	1:200	Cell Signaling Technology, Inc. USA. cat. # 19589T

Table S2. List of secondary fluorescent antibodies

Secondary detection system used	Host	Dilution	Supplier
Anti-Rabbit-IgG (H+L)-488	Goat	1:500	Proteintech Group, China, Cat. # SA00013-2
Anti-Mouse-IgG (H+L)-488	Goat	1:200	Proteintech Group, China, Cat. # SA00013-1

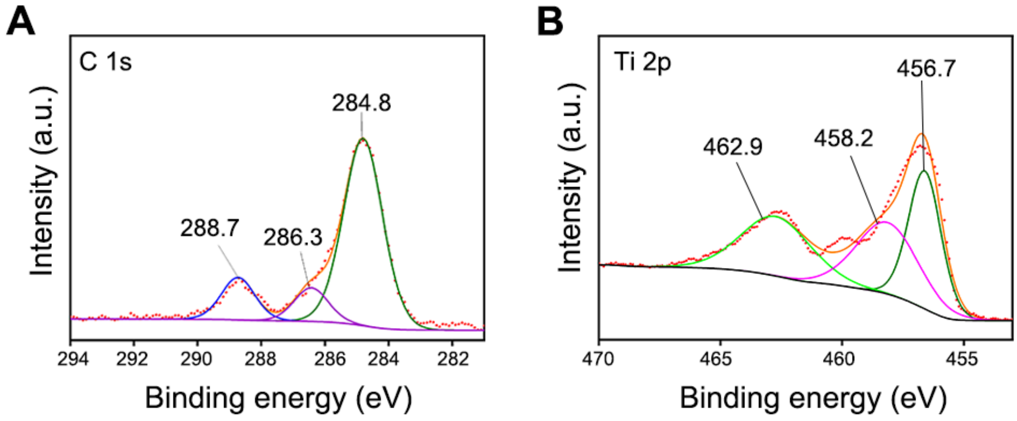

Fig. S1. XPS spectrum of MCe heterojunction, including C 1s (**A**) and Ti 2p (**B**).

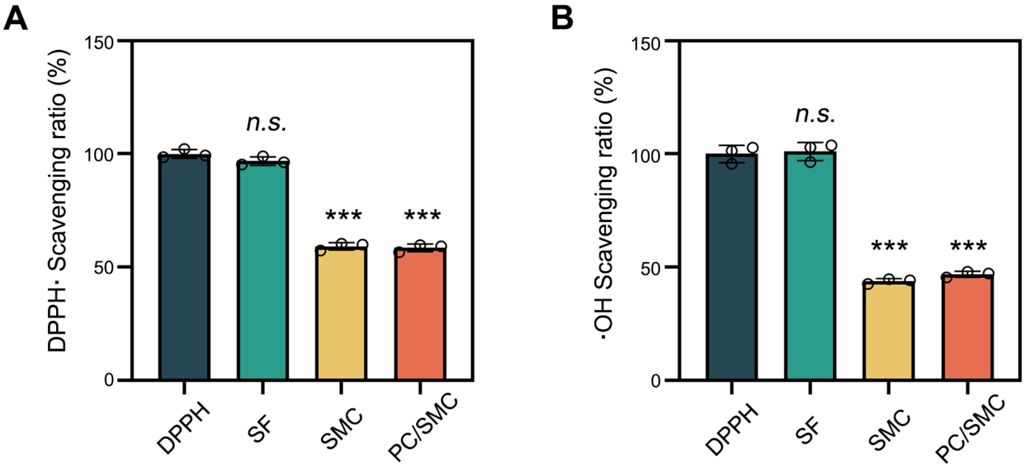

Fig. S2. (**A**) Quantitative results of DPPH assay (n = 3); (**B**) Quantitative results of TMB assay (n = 3). Values are shown as the Mean ± SD. For the significant comparison, *n.s.* indicates no significance, ****P* < 0.001.

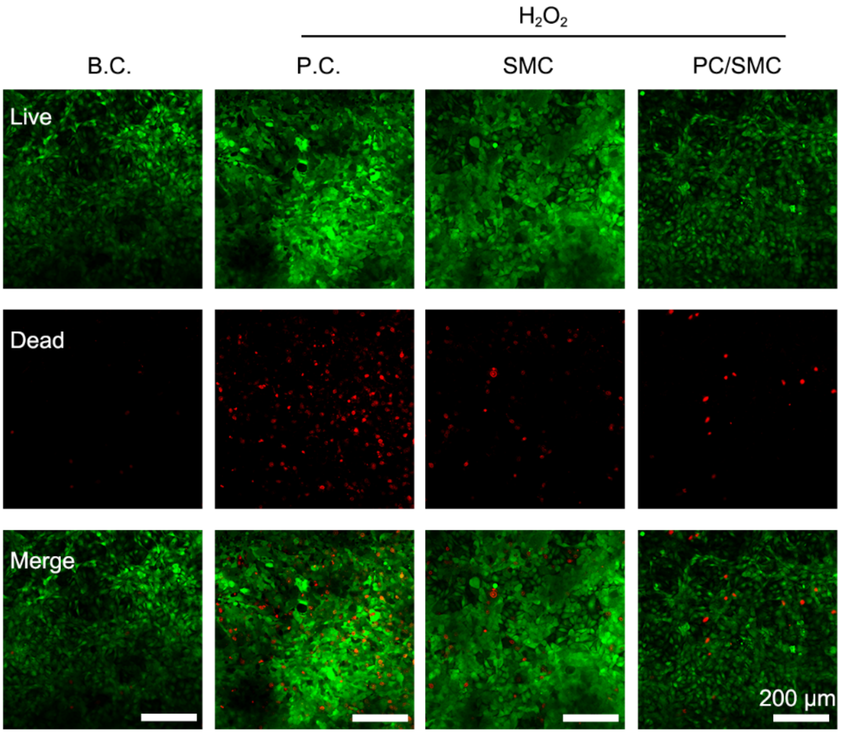

Fig. S3. Live/dead cell staining images of HUVEC cells. Scale bar: 200 μm.

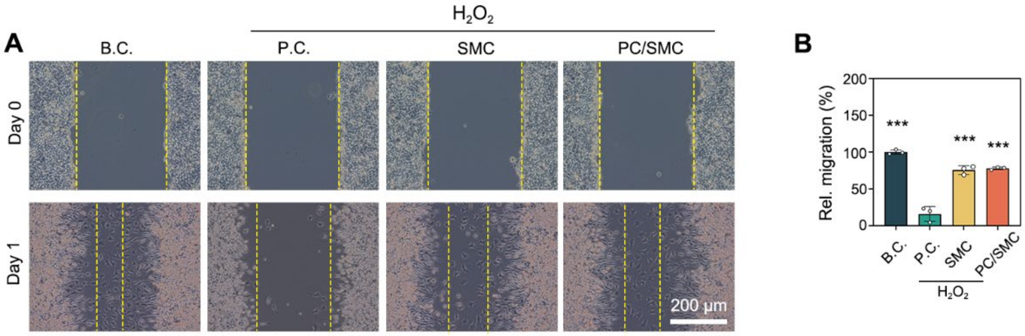

Fig. S4. (**A-B**) The migration ability of L929 cells were evaluated by scratching assay (n = 3). Values are shown as the Mean ± SD. Compared to P.C. group, ***$P < 0.001$.

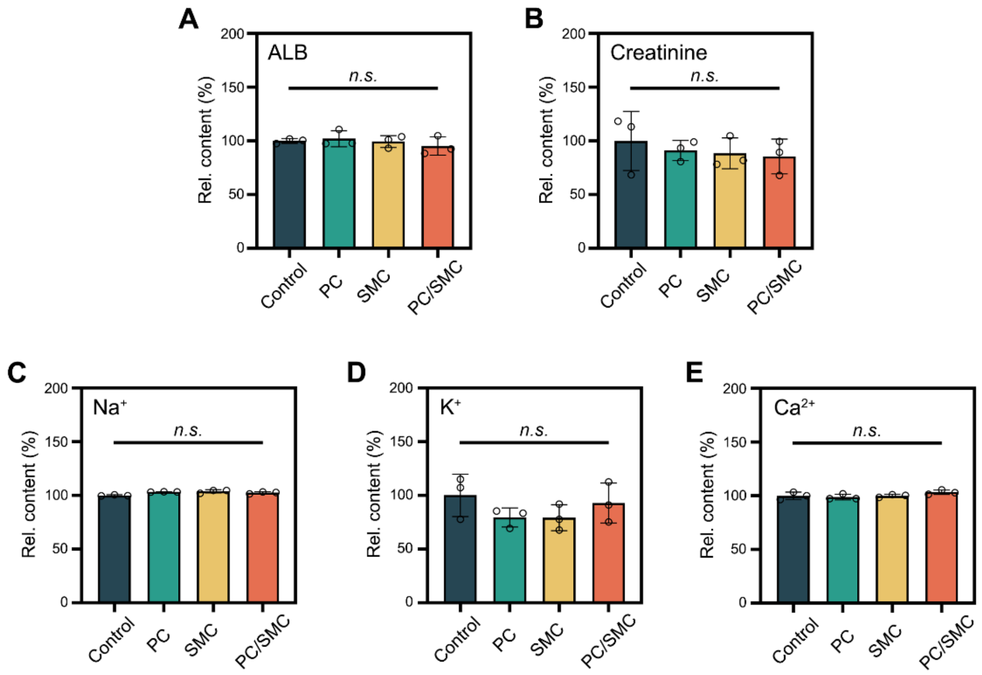

Fig. S5. Blood biochemical tests, including (**A**) albumin (ALB), (**B**) creatinine, (**C**) Na^+, (**D**) K^+ and (**E**) Ca^{2+} (n = 3). Values are shown as the Mean ± SD. Compared to P.C. group, *n.s.* indicating no significant.

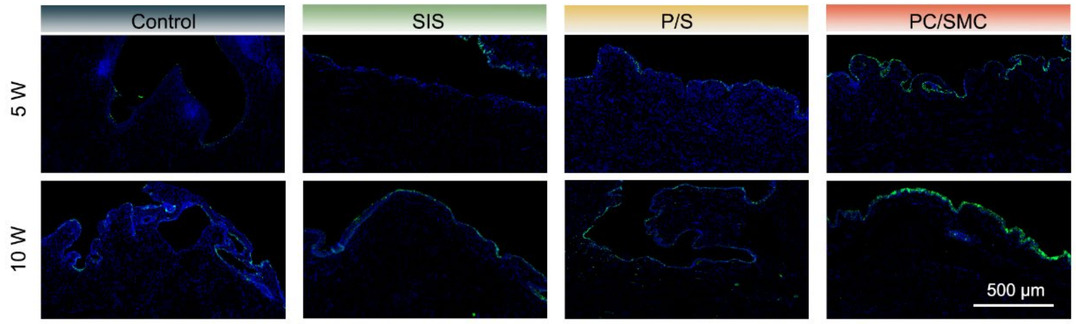

Fig. S6. IF staining images of AE1/AE3. Scale bar: 500 μm.

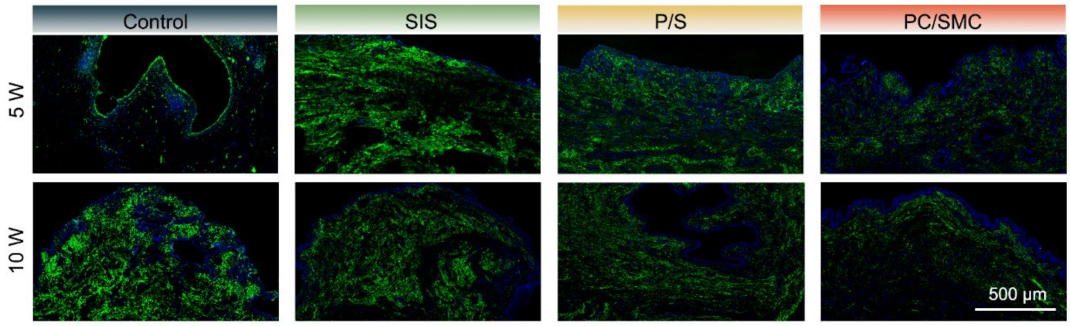

Fig. S7. IF staining images of TGF-β. Scale bar: 500 μm.

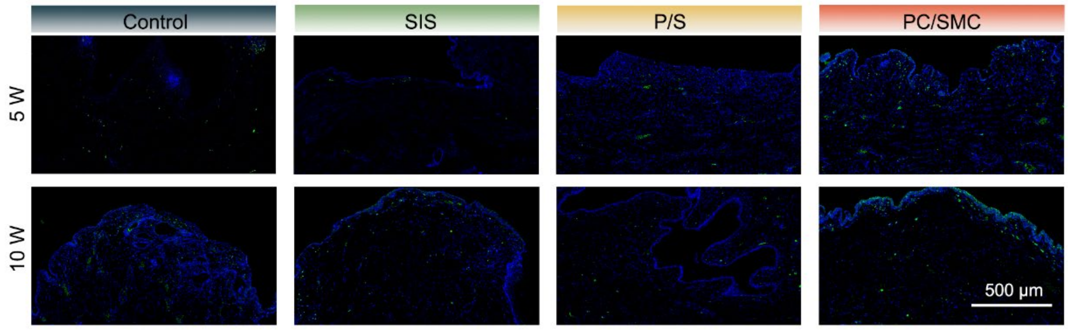

Fig. S8. IF staining images of Ki-67. Scale bar: 500 μm.

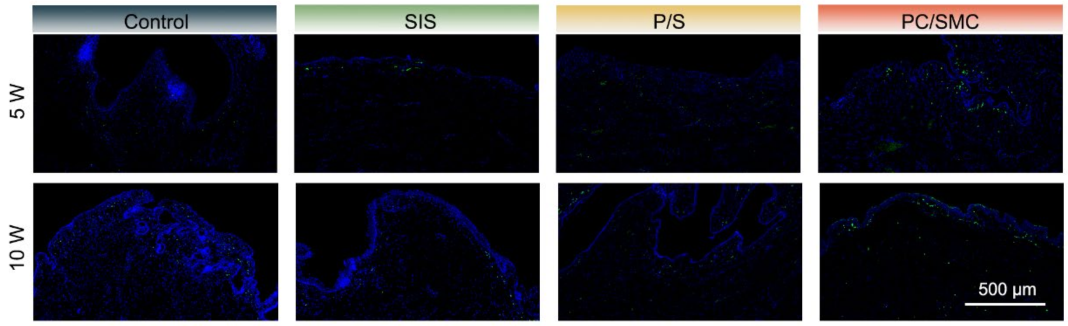

Fig. S9. IF staining images of CD206. Scale bar: 500 μm.

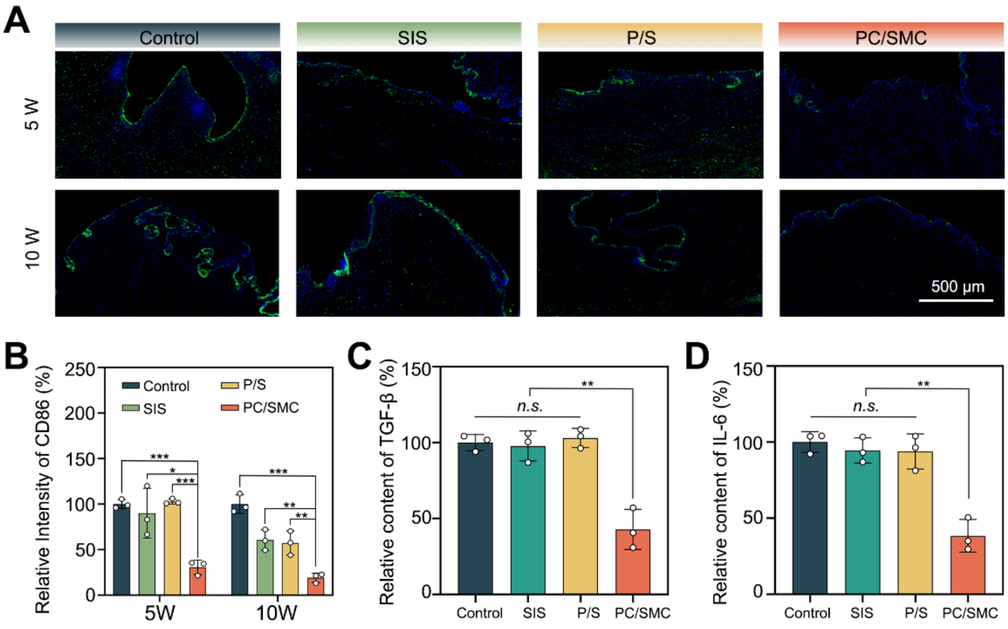

Fig. S10. (**A**) IF staining images of CD86. Scale bar: 500 μm; (**B**) Quantitative results of CD86 (n = 3); (**C**) Relative content of IL-6 at Week 5 (n = 3); (**D**) Re lative content of TNF-α at Week 5 (n = 3). Values are shown as the Mean ± SD. For the significant comparison, **P* < 0.05, ***P* < 0.01, ****P* < 0.001.
